# Single-energy computed tomography-based pulmonary perfusion imaging: Proof-of-principle in a canine model

**DOI:** 10.1118/1.4953188

**Published:** 2016-06-09

**Authors:** Tokihiro Yamamoto, Michael S. Kent, Erik R. Wisner, Lynelle R. Johnson, Joshua A. Stern, Lihong Qi, Yukio Fujita, John M. Boone

**Affiliations:** Department of Radiation Oncology, University of California Davis School of Medicine, Sacramento, California 95817; Department of Surgical and Radiological Sciences, University of California Davis School of Veterinary Medicine, Davis, California 95616; Department of Medicine and Epidemiology, University of California Davis School of Veterinary Medicine, Davis, California 95616; Department of Public Health Sciences, University of California Davis, Davis, California 95616; Department of Radiation Oncology, Tokai University, Isehara, Kanagawa 259-1193, Japan; Department of Radiology, University of California Davis School of Medicine, Sacramento, California 95817

**Keywords:** lung functional imaging, pulmonary perfusion, contrast-enhanced computed tomography (CT), deformable image registration

## Abstract

**Purpose:**

Radiotherapy (RT) that selectively avoids irradiating highly functional lung regions may reduce pulmonary toxicity, which is substantial in lung cancer RT. Single-energy computed tomography (CT) pulmonary perfusion imaging has several advantages (e.g., higher resolution) over other modalities and has great potential for widespread clinical implementation, particularly in RT. The purpose of this study was to establish proof-of-principle for single-energy CT perfusion imaging.

**Methods:**

Single-energy CT perfusion imaging is based on the following: (1) acquisition of end-inspiratory breath-hold CT scans before and after intravenous injection of iodinated contrast agents, (2) deformable image registration (DIR) for spatial mapping of those two CT image data sets, and (3) subtraction of the precontrast image data set from the postcontrast image data set, yielding a map of regional Hounsfield unit (HU) enhancement, a surrogate for regional perfusion. In a protocol approved by the institutional animal care and use committee, the authors acquired CT scans in the prone position for a total of 14 anesthetized canines (seven canines with normal lungs and seven canines with diseased lungs). The elastix algorithm was used for DIR. The accuracy of DIR was evaluated based on the target registration error (TRE) of 50 anatomic pulmonary landmarks per subject for 10 randomly selected subjects as well as on singularities (i.e., regions where the displacement vector field is not bijective). Prior to perfusion computation, HUs of the precontrast end-inspiratory image were corrected for variation in the lung inflation level between the precontrast and postcontrast end-inspiratory CT scans, using a model built from two additional precontrast CT scans at end-expiration and midinspiration. The authors also assessed spatial heterogeneity and gravitationally directed gradients of regional perfusion for normal lung subjects and diseased lung subjects using a two-sample two-tailed *t*-test.

**Results:**

The mean TRE (and standard deviation) was 0.6 ± 0.7 mm (smaller than the voxel dimension) for DIR between pre contrast and postcontrast end-inspiratory CT image data sets. No singularities were observed in the displacement vector fields. The mean HU enhancement (and standard deviation) was 37.3 ± 10.5 HU for normal lung subjects and 30.7 ± 13.5 HU for diseased lung subjects. Spatial heterogeneity of regional perfusion was found to be higher for diseased lung subjects than for normal lung subjects, i.e., a mean coefficient of variation of 2.06 vs 1.59 (*p* = 0.07). The average gravitationally directed gradient was strong and significant (*R*^2^ = 0.99, *p* < 0.01) for normal lung dogs, whereas it was moderate and nonsignificant (*R*^2^ = 0.61, *p* = 0.12) for diseased lung dogs.

**Conclusions:**

This canine study demonstrated the accuracy of DIR with subvoxel TREs on average, higher spatial heterogeneity of regional perfusion for diseased lung subjects than for normal lung subjects, and a strong gravitationally directed gradient for normal lung subjects, providing proof-of-principle for single-energy CT pulmonary perfusion imaging. Further studies such as comparison with other perfusion imaging modalities will be necessary to validate the physiological significance.

## 1. INTRODUCTION

Pulmonary toxicity is substantial in lung cancer radiotherapy (RT), particularly for locally advanced disease.^1,2^ Symptomatic (grade ≥ 2) radiation pneumonitis is a common toxicity that occurs in approximately 30% of patients irradiated for lung cancer, with fatal pneumonitis in about 2% of patients.^1,2^ The current paradigm of RT is based on anatomic imaging and assumes a homogeneous radiation dose–response of normal tissues. RT that selectively avoids irradiating highly functional lung regions may reduce pulmonary toxicity. This hypothesis is supported by several reports in the literature, demonstrating that lung dose-function metrics improve predictive power for pulmonary toxicity compared to dose–volume metrics (the current clinical standard).^3–5^ In addition, information of regional lung function, such as perfusion defect, was found to be beneficial in predicting toxicity.^6,7^

There are several modalities for pulmonary perfusion imaging, including nuclear medicine imaging,^8^ magnetic resonance (MR) imaging,^9^ and dual-energy computed tomography (CT) imaging.^10^ Perfusion images can also be acquired using single-energy CT scans with deformable image registration (DIR)-assisted image subtraction, henceforth referred to as single-energy CT perfusion imaging. Single-energy CT perfusion imaging is based on the following: (1) breath-hold CT scans before and after intravenous (IV) injection of iodinated contrast agents, (2) DIR, and (3) subtraction of the precontrast image data set from the postcontrast image data set, yielding a map of regional Hounsfield unit (HU) enhancement as a surrogate for perfusion. Single-energy CT perfusion imaging has a higher spatial resolution, shorter scan time, and/or is potentially more cost-effective than other modalities. Moreover, this method has great potential for widespread clinical implementation, particularly for applications in RT, considering that single-energy CT is already available at most RT centers and that many lung cancer patients (especially those with centrally located tumors) receive CT scans with IV contrast for improved target delineation.^11^ Some of these routinely acquired CT image data could be used for perfusion imaging. Single-energy CT perfusion imaging was first proposed by Wildberger *et al.* using a swine model with artificially induced pulmonary embolism.^12^ Their method was based on precontrast and postcontrast CT scans acquired during a single breath-hold (<30 s), and they successfully visualized perfusion defects. However, a long breath-hold poses a challenge for clinical implementation of this method, as many patients, especially lung cancer patients with poor pulmonary function, cannot tolerate holding their breath for extended periods.

The purpose of this study was to establish proof-of-principle for single-energy CT perfusion imaging based on precontrast and postcontrast CT scans acquired with two separate breath-holds using canine models of normal lungs and diseased lungs. The accuracy of DIR between the precontrast and postcontrast CT image data sets was quantified, as this is the key to accurate computation of regional perfusion. Moreover, we assessed spatial heterogeneity and gravitationally directed gradients of regional perfusion for normal lung subjects and diseased lung subjects.

## 2. METHODS AND MATERIALS

### 2.A. Subjects

We used seven canines that were considered to have normal lungs (no clinical or radiographic evidence of pulmonary disease) and seven canines with diseased lungs (including primary lung tumor, lung metastasis, and bronchointerstitial pneumonia) recruited from animals presenting to the William R. Pritchard Veterinary Medical Teaching Hospital at University of California Davis for veterinary care. Dogs had to be at least one year of age, weigh between 10 and 50 kg, and have adequate health and organ function as identified on a physical examination, complete blood count, and a chemistry panel to allow safe anesthesia administration. Owner informed consent was obtained. The protocol was approved by the institutional clinical trials review board and the institutional animal care and use committee.

Dogs were premedicated with intramuscular administration of either butorphanol (0.3 mg/kg) or hydromorphone (0.05 mg/kg) and atropine (0.02 mg/kg). An IV catheter was placed in a cephalic vein. Anesthesia was induced with propofol (2 mg/kg IV) and midazolam (0.1 mg/kg IV) to effect in most cases. Dogs were intubated and maintained on isoflurane gas anesthesia to effect. An arterial catheter was placed in the dorsal pedal artery. Dogs were kept in ventral recumbency to limit the effects of atelectasis. Heart rate, blood pressure, body temperature, depth of anesthesia, and end-tidal carbon dioxide were monitored throughout the anesthetic procedure. The dogs were then transported to the CT scanner and kept in ventral recumbency when placed on the CT table.

### 2.B. Overview of single-energy CT perfusion imaging

**FIG. 1.**
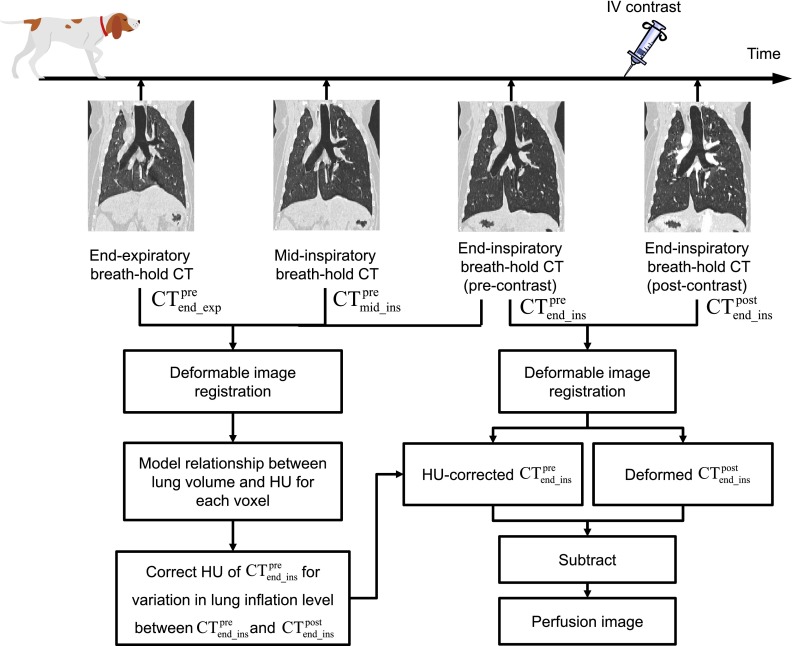
Schematic of image acquisition, processing and, analysis for single-energy CT pulmonary perfusion imaging. Three precontrast breath-hold CT scans acquired at end-expiration, midinspiration, and end-inspiration were used to correct HUs of the precontrast end-inspiratory image for variation in the lung inflation level between the precontrast and postcontrast end-inspiratory scans. A pair of the HU-corrected precontrast image data set and deformed postcontrast image data set was used for perfusion computation.

Figure 1 shows a schematic of image acquisition, processing, and analysis for single-energy CT pulmonary perfusion imaging. End-inspiratory breath-hold CT scans before and after IV injection of iodinated contrast (${\text{CT}}_{\text{end}\_\text{ins}}^{\text{pre}}$ and ${\text{CT}}_{\text{end\_ins}}^{\text{post}}$, respectively) were acquired for perfusion computation. In addition, two CT scans were also acquired at end-expiration (${\text{CT}}_{\text{end\_exp}}^{\text{pre}}$) and midinspiration (${\text{CT}}_{\text{mid\_ins}}^{\text{pre}}$) before contrast administration to build a model to correct HUs of ${\text{CT}}_{\text{end\_ins}}^{\text{pre}}$ for variation in the lung inflation level between the ${\text{CT}}_{\text{end\_ins}}^{\text{pre}}$ and ${\text{CT}}_{\text{end\_ins}}^{\text{post}}$ scans. The main reason of using end-inspiratory breath-hold CT scans for perfusion computation was because this study was designed based on a likely clinical scenario in RT applications. End-inspiratory breath-hold CT scans are sometimes preferred rather than end-expiratory CT scans because of a relatively long acquisition time for the whole lungs on CT scanners currently available at most RT centers than on diagnostic CT scanners.

### 2.C. Breath-hold CT imaging

Breath-hold CT scans were acquired in the prone position with a LightSpeed 16-slice CT scanner (GE Healthcare, Waukesha, WI). Assisted hyperventilation was performed before CT scans to facilitate breath-hold. Scan parameters were as follows: 120 kV, 150 mA, 1.25 mm slice thickness, 1 s rotation time, and 1.375 pitch. For ${\text{CT}}_{\text{end\_ins}}^{\text{post}}$ scans, iodinated contrast (3 ml/kg; Isovue, 370 mg of iodine/ml, Bracco Diagnostics, Cranbury Township, NJ) was administered intravenously at a flow rate of 4 ml/s through a peripheral IV line inserted into the cephalic vein. The delay time between the start of contrast administration and start of CT scan ranged from 13 to 20 s, depending on the volume of contrast agent administered. CT images were reconstructed using a filtered backprojection algorithm with a sharp reconstruction kernel (GE Lung).

### 2.D. Deformable image registration

DIR for spatial mapping of ${\text{CT}}_{\text{end\_ins}}^{\text{post}}$ (moving) to ${\text{CT}}_{\text{end\_ins}}^{\text{pre}}$ (fixed) was performed for perfusion computation (Fig. 1). DIR of ${\text{CT}}_{\text{end\_exp}}^{\text{pre}}$ (moving) to ${\text{CT}}_{\text{mid\_ins}}^{\text{pre}}$ (fixed) and then ${\text{CT}}_{\text{mid\_ins}}^{\text{pre}}$ (moving) to ${\text{CT}}_{\text{end\_ins}}^{\text{pre}}$ (fixed) was also performed to correct HUs of ${\text{CT}}_{\text{end\_ins}}^{\text{pre}}$ for variation in the lung inflation level (see Sec. 2.E). elastix, an open source software package for medical image registration,^13^ was used along with parameter settings used by Metz *et al.*^14^ B-spline DIR was performed in a multigrid setting, which was driven by a similarity function and a transform bending energy penalty (set to 0.05 for all conditions).^15^ For the similarity function, normalized cross correlation and mutual information were compared to select the one that showed better performance based on visual assessment. Normalized cross correlation was selected for DIR between ${\text{CT}}_{\text{end\_ins}}^{\text{post}}$ and ${\text{CT}}_{\text{end\_ins}}^{\text{pre}}$, and mutual information for DIR between ${\text{CT}}_{\text{end\_exp}}^{\text{pre}}$ and ${\text{CT}}_{\text{mid\_ins}}^{\text{pre}}$, and between ${\text{CT}}_{\text{mid\_ins}}^{\text{pre}}$ and ${\text{CT}}_{\text{end\_ins}}^{\text{pre}}$. For the image data, Gaussian pyramids were used for down-sampling to increase robustness. For the B-spline transformation, a multigrid approach was used. Five resolution levels were used with the following parameter settings: down-sampling factor 16, 8, 4, 2, and 1; B-spline grid spacing 80, 40, 20, 10, and 5 mm. DIR was performed with lung masks that were generated by segmenting the lungs on both the fixed and moving CT images and merging those segmented lungs together. The DIR method and parameter settings used in this study are similar to Staring *et al.*^16^

The accuracy of DIR was evaluated based on the target registration error (TRE) of anatomic pulmonary landmarks and on singularities in the displacement vector field (DVF) in a manner similar to the MICCAI EMPIRE10 Challenge.^17^ The TRE is defined as the distance between landmarks in the fixed image mapped from the moving image by DIR and those mapped manually as the reference. Manual annotation of landmarks was performed by a medical physicist. We used the iX software^18^ to generate 50 landmarks per subject distributed throughout the lungs. The DVF was also analyzed for singularities, i.e., regions where the DVF is not bijective. The Jacobian determinant of the DVF was calculated for each voxel and analyzed to examine whether there was any voxel with a negative Jacobian value. The following two scenarios of DIR were evaluated for 10 randomly selected subjects out of 14 subjects: (1) ${\text{CT}}_{\text{end\_exp}}^{\text{pre}}$ (moving) to ${\text{CT}}_{\text{mid\_ins}}^{\text{pre}}$ (fixed), and (2) ${\text{CT}}_{\text{end\_ins}}^{\text{post}}$ (moving) to ${\text{CT}}_{\text{end\_ins}}^{\text{pre}}$ (fixed).

### 2.E. HU correction for variation in lung inflation level

Lung HUs vary with the lung inflation level (depth of breathing), i.e., lower HUs for deeper breathing. Thus, the lung inflation variation between ${\text{CT}}_{\text{end\_ins}}^{\text{pre}}$ and ${\text{CT}}_{\text{end\_ins}}^{\text{post}}$ hampers accurate computation of postcontrast HU enhancement. To address this issue, we corrected HUs of ${\text{CT}}_{\text{end\_ins}}^{\text{pre}}$ for the lung inflation variation based on a relationship between the HU for each voxel and total lung volume as a measure of the lung inflation level. This relationship was determined using three precontrast CT scans (${\text{CT}}_{\text{end\_exp}}^{\text{pre}}$, ${\text{CT}}_{\text{mid\_ins}}^{\text{pre}}$, and ${\text{CT}}_{\text{end\_ins}}^{\text{pre}}$) as follows. First, DIR was performed to establish the spatial correspondence between the three image data sets for each voxel. Second, a relationship between the HU and total lung volume (measured by segmented lungs in the CT image) was modeled using linear regression for each voxel. Third, this relationship was used to estimate a HU at the lung inflation level of ${\text{CT}}_{\text{end\_ins}}^{\text{post}}$ by linear interpolation or extrapolation. Finally, the original HU of ${\text{CT}}_{\text{end\_ins}}^{\text{pre}}$ was replaced with the estimated HU for each voxel, yielding an image with precontrast HUs at the same lung inflation level as ${\text{CT}}_{\text{end\_ins}}^{\text{post}}$. For perfusion computation, the resulting HU-corrected ${\text{CT}}_{\text{end\_ins}}^{\text{pre}}$ image data set was subtracted from the deformed ${\text{CT}}_{\text{end\_ins}}^{\text{post}}$ image data set.

### 2.F. Analysis of CT perfusion images

Spatial heterogeneity of regional perfusion was evaluated to determine whether diseased lung subjects demonstrated higher heterogeneity than normal lung subjects. Several investigators reported significantly higher heterogeneity of regional perfusion or ventilation for subjects with diseased lungs than healthy subjects.^19,20^ The coefficient of variation (CoV), i.e., standard deviation (SD)/mean of HU enhancement was used to quantify overall heterogeneity of regional perfusion. The CoV has been used by several investigators in the literature.^19–22^ Lung masks were generated by delineating voxels with less than −250 HU within lung outlines. A two-sample two-tailed *t*-test was used to compare the CoVs of diseased lung subjects with those of normal lung subjects.

Moreover, gravitationally directed gradients of regional perfusion were evaluated to determine whether single-energy CT perfusion imaging demonstrated the known effect of gravity on regional perfusion, i.e., greater perfusion in gravity-dependent (ventral) regions than in nondependent (dorsal) regions. This effect has been demonstrated with other imaging modalities.^22–24^ The slope (regression coefficient) was quantified from linear regression for the relationship between the relative ventral-to-dorsal distance and HU enhancement. The total lung was divided into five coronal section regions of interest (ROIs), equally spaced along the ventral-to-dorsal direction. The mean HU enhancement was calculated for each ROI. Statistical analysis was performed to test whether the slope was significantly different from zero (*p* < 0.05) using a two-sample two-tailed *t*-test.

## 3. RESULTS

### 3.A. Accuracy of deformable image registration

**FIG. 2.**
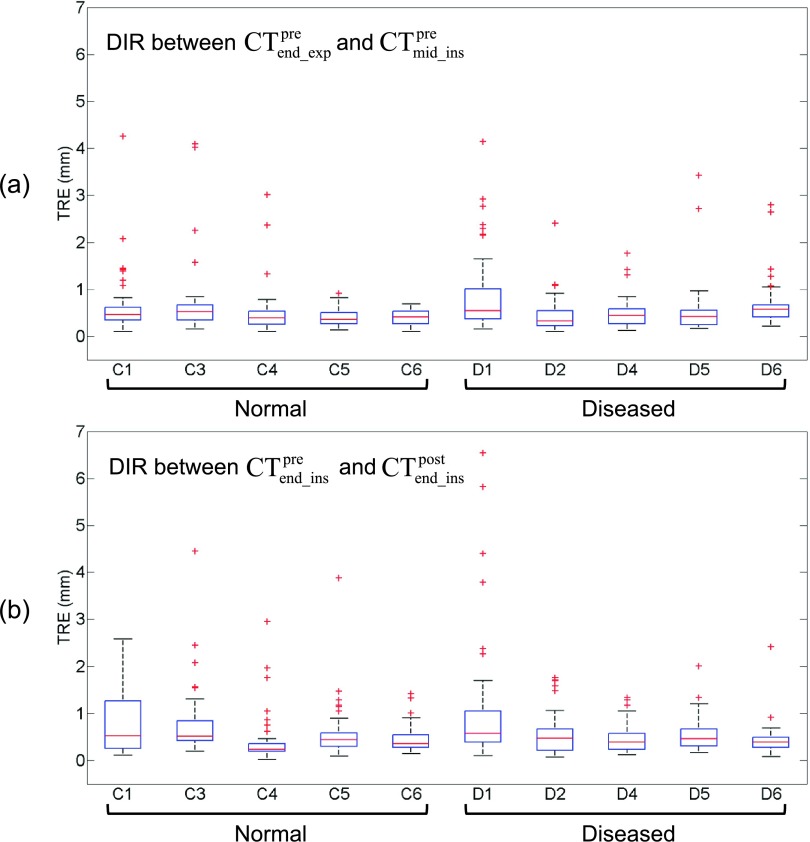
Box plots of target registration errors (TREs) for DIR (a) between end-expiratory (${\text{CT}}_{\text{end\_exp}}^{\text{pre}}$) and midinspiratory CT image data sets (${\text{CT}}_{\text{mid\_ins}}^{\text{pre}}$), and (b) between precontrast end-inspiratory (${\text{CT}}_{\text{end\_ins}}^{\text{pre}}$) and postcontrast end-inspiratory CT image data sets (${\text{CT}}_{\text{end\_ins}}^{\text{post}}$) for five normal lung subjects and five diseased lung subjects (50 pulmonary landmarks/subject). Each box is composed of three horizontal lines corresponding to the 25th, 50th (median), and 75th percentile. Whiskers extend to 2.7 times the standard deviation. Outliers are plotted as individual points.

Across the entire data set, the mean TRE (and SD) was 0.6 ± 0.6 mm for DIR between ${\text{CT}}_{\text{end\_exp}}^{\text{pre}}$ and ${\text{CT}}_{\text{mid\_ins}}^{\text{pre}}$, and 0.6 ± 0.7 mm for DIR between ${\text{CT}}_{\text{end\_ins}}^{\text{pre}}$ and ${\text{CT}}_{\text{end\_ins}}^{\text{post}}$. The mean TRE was smaller than the voxel dimension (approximately 1 × 1 × 1.25 mm^3^) in both DIR scenarios. Figure 2 shows a box plot of TREs for individual subjects. For most cases, there were only several landmarks with a TRE exceeding the voxel dimension. Subject C1 and D1 showed a relatively large number (range 6–13) of such TREs in both DIR scenarios. In particular, subject D1 showed large TREs of up to 6.5 mm, many of which occurred around dark streaks of beam hardening artifacts from dense iodinated contrast in great vessels. Table I shows weight and lung volumes of four breath-hold CT scans for individual subjects. The % differences in the lung volume ranged from 11.2% to 67.1% for the pair of ${\text{CT}}_{\text{end\_exp}}^{\text{pre}}$ and ${\text{CT}}_{\text{mid\_ins}}^{\text{pre}}$, and 0.2%–16.0% for the pair of ${\text{CT}}_{\text{end\_ins}}^{\text{pre}}$ and ${\text{CT}}_{\text{end\_ins}}^{\text{post}}$. There were only weak correlations between the maximum TREs and % differences in the lung volume for both pairs: ${\text{CT}}_{\text{end\_exp}}^{\text{pre}}$ and ${\text{CT}}_{\text{mid\_ins}}^{\text{pre}}$ (*r* = 0.27), ${\text{CT}}_{\text{end\_ins}}^{\text{pre}}$ and ${\text{CT}}_{\text{end\_ins}}^{\text{post}}$ (*r* = 0.50). No singularities were observed in the displacement vector fields for both DIR scenarios.

### 3.B. Overall HU enhancement

**FIG. 3.**
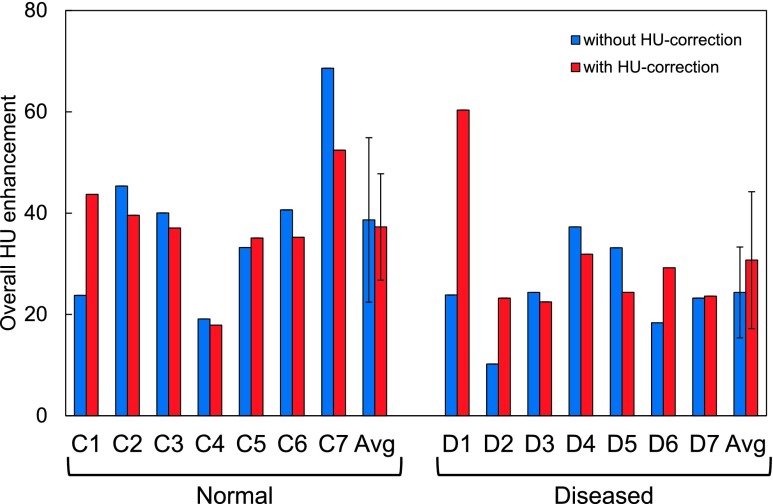
Overall postcontrast HU enhancement (average in the lung parenchyma) without and with HU correction of precontrast CT images for seven normal lung subjects and seven diseased lung subjects.

Figure 3 shows the overall postcontrast HU enhancement without and with HU correction of the precontrast CT images for individual subjects. With HU correction, the mean enhancement (and SD) was 37.3 ± 10.5 HU for normal lung subjects and 30.7 ± 13.5 HU for diseased lung subjects. There was no significant difference between the normal and diseased lung groups (*p* = 0.33). The HU enhancement varied widely among subjects, ranging from 17.9 HU (C4) to 60.3 HU (D1).

### 3.C. CT perfusion images

**FIG. 4.**
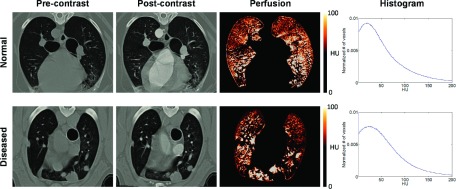
Example images of the precontrast (${\text{CT}}_{\text{end\_ins}}^{\text{pre}}$) (HU-corrected), postcontrast (${\text{CT}}_{\text{end\_ins}}^{\text{post}}$) (deformed to precontrast) and perfusion for two representative subjects: normal (C6) and diseased (D4). Histograms of perfusion (HU enhancement) are also shown. Subject C6 showed a more homogeneous perfusion distribution with a more sharp-peaked histogram and a lower coefficient of variation (CoV) (1.66) compared to subject D4 (2.10).

Figure 4 shows example images of ${\text{CT}}_{\text{end\_ins}}^{\text{pre}}$, deformed ${\text{CT}}_{\text{end\_ins}}^{\text{post}}$, and perfusion for two representative subjects: normal (C6) and diseased (D4). The ${\text{CT}}_{\text{end\_ins}}^{\text{post}}$ images showed considerable HU enhancement in great vessels and slight enhancement in the lung parenchyma. The perfusion image of subject C6 showed a more homogeneous distribution (CoV = 1.66) and more sharply peaked histogram compared to subject D4 showing poorly perfused regions near the tumors (CoV = 2.10). Subject C6 showed higher perfusion in gravity-dependent ventral regions than in nondependent regions.

Table II shows CoVs for individual subjects. The mean CoV of the normal lung group was 1.59, which was lower (i.e., more homogeneous) than the diseased lung group (mean CoV = 2.06) (*p* = 0.07). Although the difference was not statistically significant at the 0.05 level, this result suggests that spatial heterogeneity of regional perfusion is higher for diseased lung subjects than for normal lung subjects.

**FIG. 5.**
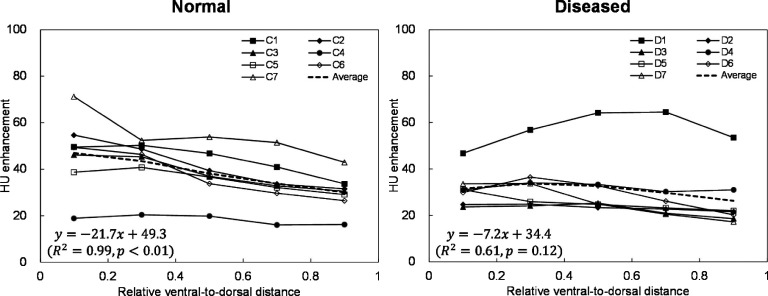
Ventral-to-dorsal gradients of regional perfusion for seven normal lung dogs, seven diseased lung dogs, and average for each group. Each data point represents the mean HU enhancement in a coronal section ROI. The equations from linear regression are also shown for the average gradients.

Figure 5 shows ventral-to-dorsal gradients of regional perfusion for individual subjects as well as an average for each group. The average ventral-to-dorsal gradient of the normal lung group was found to be strong and significant (*R*^2^ = 0.99, *p* < 0.01), indicating higher perfusion in gravity-dependent ventral regions than in nondependent regions. In contrast, the diseased lung group showed a moderate, nonsignificant gradient (*R*^2^ = 0.61, *p* = 0.12). The *R*^2^ values ranged from 0.57 (subject C4) to 0.96 (subject C2) for normal lung subjects, and 0.12 (subject D4) to 0.94 (subject D7) for diseased lung subjects.

## 4. DISCUSSION

This canine study on single-energy CT pulmonary perfusion imaging demonstrated the accuracy of DIR with subvoxel TREs on average, higher spatial heterogeneity of regional perfusion for diseased lung subjects than for normal lung subjects, and a strong gravitationally directed gradient for normal lung subjects. These results provide proof-of-principle for single-energy CT perfusion imaging. This is the first investigation to quantify spatial heterogeneity and a gravitationally directed gradient using single-energy CT perfusion imaging. Our results were consistent with the previous studies based on other imaging modalities. Vidal Melo *et al.* demonstrated higher heterogeneity of regional perfusion measured by ^13^N positron emission tomography (PET) for patients with chronic obstructive pulmonary disease (COPD) compared to healthy subjects.^20^ Similarly, Tzeng *et al.* showed higher ventilation heterogeneity measured by hyperpolarized ^3^He MRI for asthmatic patients than healthy subjects.^19^ The gravitationally directed gradient of regional perfusion has also been reported by many investigators with several different imaging modalities including ^99m^Tc-labeled macroaggregated albumin (MAA) single-photon emission CT (SPECT),^23^ Fourier decomposition MRI,^24^ and arterial spin labeling MRI.^22^ High heterogeneity in diseased lungs and a ventral-to-dorsal gradient in normal lungs are both necessary but not sufficient conditions for accurate perfusion imaging. Further studies such as a comparison with other perfusion imaging modalities will be necessary for validation in the future.

The proposed method of single-energy CT perfusion imaging is considered a form of parametric response mapping (PRM), an emerging method of classifying and measuring tissue changes voxel by voxel. PRM has been used in oncology for assessment of response to therapy for gliomas,^25^ head and neck cancer,^26^ breast cancer,^27^ and metastatic prostate cancer to the bone,^28^ and moreover for assessment of COPD phenotypes.^29^ For example, a PRM parameter, i.e., % of voxels within the tumor yielding increased apparent diffusion coefficient (ADC) values determined by pretreatment and midtreatment diffusion-weighted MRI, was found to be predictive of clinical progression for head and neck cancer patients, whereas % changes in tumor volume and whole-tumor ADC were not predictive.^26^ Similar analysis to investigate the predictive power of single-energy CT perfusion imaging for pulmonary toxicity after RT or respiratory diseases would be a fascinating topic for future work.

A pair of the end-expiration and midinspiration or end-inspiration breath-hold CT scans acquired for HU correction can also be used to compute regional volume change, a surrogate for ventilation.^30–33^ Under normal conditions, regional ventilation and perfusion are tightly matched to each other, yielding efficient gas exchange. However, normal distributions of regional ventilation and perfusion change dramatically under pathological conditions, leading to ventilation/perfusion mismatch and inefficient gas exchange.^34^ For example, Vidal Melo *et al.* reported significantly higher heterogeneity of ventilation/perfusion ratios measured by ^13^N PET for sheep with pulmonary embolism, saline lung lavage or bronchoconstriction compared to control sheep.^35^ Investigations into ventilation and perfusion, e.g., comparison of ventilation/perfusion heterogeneity in diseased lungs and in normal lungs are currently underway to provide further validation of CT perfusion imaging.

**FIG. 6.**
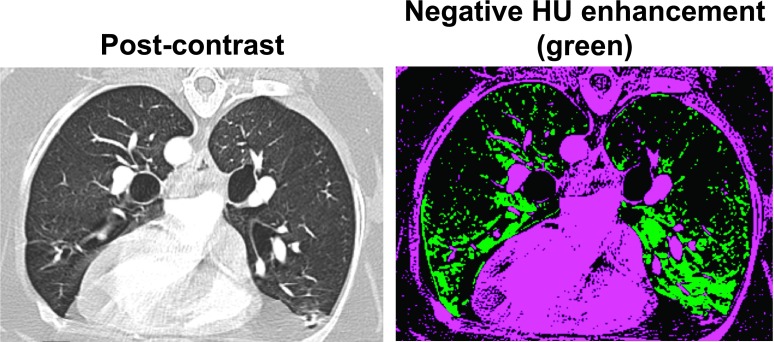
Example images of the postcontrast CT (left) and voxels with negative HU enhancement denoted in green (right) for subject D1. The number of voxels with negative HU enhancement accounted for 15% of the lung voxels. (See color online version.)

There are several limitations in this study. First, dense concentration of iodinated contrast in great vessels caused dark streaks (beam hardening artifacts), which could lead to DIR errors and negative HU enhancement due to lower HUs in the postcontrast CT image. Voxels with negative HU enhancement appeared to be distributed mainly in the proximity of great vessels as shown in Fig. 6. There were a considerable number of voxels with negative HU enhancement, perhaps because of beam hardening artifacts and also residual DIR errors and/or uncertainty in the HU correction method for precontrast CT images. For future studies, strategies that reduce beam hardening artifacts such as an iterative reconstruction algorithm^36^ might increase the accuracy of DIR and reduce negative HU enhancement. Second, the HU enhancement was relatively subtle (34 HU on average) and varied widely among subjects (range 17.9–60.3 HU), suggesting that there is room for improvement. The average HU enhancement of 34 HU in this study is similar to the work of Groell *et al.*, which observed an enhancement of 30 HU in the normal lung segment by comparing precontrast and postcontrast CT scans.^37^ Also the 13–20 s delay time between contrast administration and CT image acquisition in this study is similar to other CT perfusion studies, e.g., 13 s by Wildberger *et al.*^12^ and 15 s by Fuld *et al.*,^38^ Given that this study included dogs with varying breeds (e.g., Pembroke Welsh Corgi and Shepherd), weight (range 13.1–42.5 kg), and lung pathologies (normal, primary lung tumor, lung metastasis, or interstitial lung disease), there might be intersubject variability in the hemodynamic profile, which could influence the optimal delay time. Optimizing the delay time using a more specific patient population would be an important subject of future work. Finally, different disease models were used in this study, which might have contributed to a nonsignificant difference in the mean CoV between the normal and diseased lung groups (*p* = 0.07). Some dogs had relatively small lung tumors that might have little effect on regional perfusion. For future studies, respiratory diseases that result in large perfusion defects (e.g., pulmonary embolism) may provide further insights into the physiological significance of CT perfusion imaging. Also other methods to quantify heterogeneity, including texture analysis, fractal techniques, and Minkowski functionals, should also be considered.

Table III shows advantages and disadvantages of pulmonary perfusion imaging modalities including single-energy CT, dual-energy CT, SPECT, and MRI. One of the major disadvantages of single-energy CT perfusion imaging is that multiple CT acquisitions (i.e., precontrast and postcontrast CT, and CT at different lung inflation levels for HU correction) are required for accurate perfusion computation, leading to a higher radiation dose than other modalities. This disadvantage may not pose a major challenge for applications in RT. Most patients treated with modern RT receive CT scans for treatment planning. Also many lung cancer patients (especially centrally located tumors^11^) receive CT scans with IV contrast for improved target delineation on a routine basis. These treatment planning CT scans could also serve as a precontrast or postcontrast CT for perfusion imaging. Furthermore, four-dimensional (4D) CT acquired for motion management is estimated to be used at approximately 70% of RT centers in the US.^39^ A HU correction model could also be built from the 4D CT image data. Nevertheless, strategies for dose reduction and optimization should be explored prior to clinical applications.

## 5. CONCLUSIONS

This canine study demonstrated the accuracy of DIR with subvoxel TREs on average, higher spatial heterogeneity of regional perfusion for diseased lung subjects than normal lung subjects, and a strong ventral-to-dorsal gradient for normal lung subjects, providing proof-of-principle for single-energy CT pulmonary perfusion imaging. Further studies such as comparison with other perfusion imaging modalities (e.g., ^99m^Tc − MAA SPECT) will be necessary to validate the physiological significance.

## Figures and Tables

**TABLE I.** Weight and lung volumes of four breath-hold CT scans for seven normal lung subjects and seven diseased lung subjects.

	Lung volume (cm^3^)					% difference	
Subject	Weight (kg)	${\text{CT}}_{\text{end\_exp}}^{\text{pre}}$	${\text{CT}}_{\text{mid\_ins}}^{\text{pre}}$	${\text{CT}}_{\text{end\_ins}}^{\text{pre}}$	${\text{CT}}_{\text{end\_ins}}^{\text{post}}$	$\left({\text{CT}}_{\text{mid\_ins}}^{\text{pre}}-{\text{CT}}_{\text{end\_exp}}^{\text{pre}}\right)/{\text{CT}}_{\text{end\_exp}}^{\text{pre}}$	$\left({\text{CT}}_{\text{end\_ins}}^{\text{post}}-{\text{CT}}_{\text{end\_ins}}^{\text{pre}}\right)/{\text{CT}}_{\text{end\_ins}}^{\text{pre}}$
Normal							
C1	42.5	2217	3193	3532	3888	44.0	10.1
C2	13.1	302	468	514	481	54.7	−6.3
C3	25.5	963	1610	1732	1671	67.1	−3.5
C4	22.0	1592	1847	2059	2029	16.0	−1.4
C5	34.4	2090	2524	3075	3085	20.8	0.3
C6	36.4	1534	2330	2422	2316	51.9	−4.4
C7	24.4	792	1084	1137	1032	37.0	−9.3
Mean	28.3	1356	1865	2067	2072	41.7	−2.1
Diseased							
D1	40.4	920	1296	1764	2046	40.9	16.0
D2	13.7	906	1212	1202	1295	33.8	7.7
D3	33.6	1533	2035	2294	2247	32.7	−2.0
D4	13.5	616	726	824	801	17.9	−2.7
D5	23.0	1468	1677	1959	1847	14.2	−5.7
D6	26.6	1489	1922	2060	2184	29.1	6.0
D7	21.2	968	1076	1171	1169	11.2	−0.2
Mean	24.6	1128	1420	1610	1656	25.7	2.7

**TABLE II.** CoVs as a measure of overall heterogeneity of regional perfusion for seven normal lung dogs and seven diseased lung dogs.

Subject	CoV
Normal	
C1	1.48
C2	1.14
C3	1.35
C4	2.44
C5	1.73
C6	1.66
C7	1.34
Mean	1.59
Diseased	
D1	1.22
D2	2.36
D3	2.02
D4	2.10
D5	2.06
D6	1.97
D7	2.67
Mean	2.06

**TABLE III.** Advantages and disadvantages of pulmonary perfusion imaging modalities.

Modality	Advantages	Disadvantages
Single-energy CT	Excellent availability of single-energy CT scanners in RT centers, great potential for widespread implementation, high resolution, and applicability to RT (large bore; electron density for dose calculation)	Multiple acquisitions required, DIR optimization and validation required, radiation dose, and potential side effects of iodinated contrast agents
Dual-energy CT	High resolution, applicability to RT (electron density for dose calculation)	Limited availability in RT centers, virtual noncontrast imaging may not satisfactory remove the need for multiple acquisitions (Ref. 40), small FOV for dual-source CT, typically ineffective in obese patients, radiation dose, potential side effects of iodinated contrast agents, and typically small bore
SPECT	Well-established	Limited resolution, limited availability in RT centers, and radiation dose
MRI	No radiation dose and excellent soft tissue contrast	Limited availability in RT centers and potential side effects of gadolinium-based contrast agents (dynamic contrast-enhanced MRI)
